# The pandemic toll and post-acute sequelae of SARS-CoV-2 in healthcare workers at a Swiss University Hospital

**DOI:** 10.1016/j.pmedr.2022.101899

**Published:** 2022-07-08

**Authors:** Mayssam Nehme, Laure Vieux, Delphine S. Courvoisier, Olivia Braillard, Hervé Spechbach, Frederique Jacquerioz, Julien Salamun, Frederic Assal, Frederic Lador, Matteo Coen, Thomas Agoritsas, Jean-Luc Reny, Christophe Graf, Lamyae Benzakour, Riccardo Favale, Paola M. Soccal, Guido Bondolfi, Aglaé Tardin, Dina Zekry, Silvia Stringhini, Stéphanie Baggio, Stéphane Genevay, Kim Lauper, Philippe Meyer, Nana Kwabena Poku, Basile N. Landis, Marwène Grira, José Sandoval, Julien Ehrsam, Simon Regard, Camille Genecand, Garance Kopp, Ivan Guerreiro, Gilles Allali, Pauline Vetter, Laurent Kaiser, François Chappuis, Catherine Chenaud, Idris Guessous

**Affiliations:** a Division of Primary care Medicine of the Geneva University Hospitals, Geneva, Switzerland; b Division of Tropical and Humanitarian Medicine, Geneva University Hospitals, Geneva, Switzerland; c Faculty of Medicine, University of Geneva, Geneva, Switzerland; d Division of Occupational Medicine, Geneva University Hospital, Geneva, Switzerland; e Quality of Care Division, Medical Directorate, Geneva University Hospitals, Geneva, Switzerland; f Division of Infectious Diseases, Geneva University Hospitals, Geneva, Switzerland; g Geneva Center for Emerging Viral Diseases, Geneva University Hospitals, Geneva, Switzerland; h Division of Laboratory Medicine, Laboratory of Virology, Geneva University Hospitals, Geneva, Switzerland; i Cantonal Health Service, General Directorate for Health, Geneva, Switzerland; j Division of Pulmonary Medicine, Geneva University Hospitals, Geneva, Switzerland; k Division of General Internal Medicine, Geneva University Hospitals, Geneva, Switzerland; l Division of Neurology, Geneva University Hospitals, Geneva, Switzerland; m Department of Rehabilitation and Geriatrics, Geneva University Hospitals, Geneva, Switzerland; n Division of Psychiatry, Geneva University Hospitals, Geneva, Switzerland; o Division of Rheumatology, Geneva University Hospitals, Geneva, Switzerland; p Division of Cardiology, Geneva University Hospitals, Geneva, Switzerland; q Division of Otolaryngology, Geneva University Hospitals, Geneva, Switzerland; r Institute of Primary Health Care (BIHAM), University of Bern, Bern, Switzerland; s Division of Prison Health, Geneva University Hospitals, Geneva, Switzerland; t Department of Oncology, Geneva University Hospitals, Geneva, Switzerland; u Department of Medical Information Sciences, Geneva University Hospitals, Geneva, Switzerland; v Division of Emergency Medicine, Geneva University Hospitals, Geneva, Switzerland; w Leenaards Memory Center, Lausanne University Hospital, Lausanne, Switzerland

## Abstract

Healthcare workers have potentially been among the most exposed to SARS-CoV-2 infection as well as the deleterious toll of the pandemic. This study has the objective to differentiate the pandemic toll from post-acute sequelae of SARS-CoV-2 infection in healthcare workers compared to the general population.

The study was conducted between April and July 2021 at the Geneva University Hospitals, Switzerland. Eligible participants were all tested staff, and outpatient individuals tested for SARS-CoV-2 at the same hospital. The primary outcome was the prevalence of symptoms in healthcare workers compared to the general population, with measures of COVID-related symptoms and functional impairment, using prevalence estimates and multivariable logistic regression models.

Healthcare workers (n = 3083) suffered mostly from fatigue (25.5 %), headache (10.0 %), difficulty concentrating (7.9 %), exhaustion/burnout (7.1 %), insomnia (6.2 %), myalgia (6.7 %) and arthralgia (6.3 %). Regardless of SARS-CoV-2 infection, all symptoms were significantly higher in healthcare workers than the general population (n = 3556). SARS-CoV-2 infection in healthcare workers was associated with loss or change in smell, loss or change in taste, palpitations, dyspnea, difficulty concentrating, fatigue, and headache. Functional impairment was more significant in healthcare workers compared to the general population (aOR 2.28; 1.76–2.96), with a positive association with SARS-CoV-2 infection (aOR 3.81; 2.59–5.60).

Symptoms and functional impairment in healthcare workers were increased compared to the general population, and potentially related to the pandemic toll as well as post-acute sequelae of SARS-CoV-2 infection. These findings are of concern, considering the essential role of healthcare workers in caring for all patients including and beyond COVID-19.

## 1 Introduction

COVID-19 has disproportionately affected healthcare workers^1^. They have been on the frontline of this pandemic and have been constantly working when the general population was able to follow lockdown measures in order to protect themselves. Personal protective equipment and vaccination have been crucial in protecting healthcare staff from being infected (Mehta et al., 2021), however they still have been in the groups of essential workers most impacted by SARS-CoV-2 infections (Stringhini et al., 2021); (Bergwerk et al., 2021). While acute complications of SARS-CoV-2 in healthcare workers have been described in *meta*-analyses (Gholami et al., 2021), post-acute sequelae of SARS-CoV-2 (PASC) (NIH, 2021) have been less documented. The prevalence of PASC in the general population (Nehme et al., 2021, Nehme et al., 2020, Lopez-Leon et al., 2021) is potentially applicable to healthcare workers, with the additional pandemic-related toll and work-related strains on their mental and physical health, as suggested in previous pandemics (Maunder et al., 2006). A recent study in Sweden including 323 seropositive and 1072 seronegative healthcare professionals suggested that 26 % of seropositive healthcare workers still had at least one moderate to severe symptom 2 months and 15 % at 8 months after the infection versus 9 % and 3 % in seronegative healthcare workers respectively (Havervall et al., 2021). While not comparing symptoms to the general population, this study provided first-hand accounts of potential long-term symptoms in healthcare workers.

In this study, we differentiate the direct effects of SARS-CoV-2 from the pandemic-related indirect effects on healthcare workers at the Geneva University Hospitals in Geneva, Switzerland using a large sample cohort (n = 3083) and comparing results to non-healthcare workers (n = 3556) from the same source population.

## 2 Methods

From June 22, 2021 to July 1st, 2021, an online questionnaire was sent to all staff of the Geneva University Hospitals (HUG). Our definition of healthcare workers included all hospital staff. In parallel, between April 23, 2021 and July 27, 2021, the same questionnaire was sent to all individuals tested for SARS-CoV-2 at the outpatient SARS-CoV-2 testing center at the same hospital (general population). Individuals were then categorized into healthcare workers and the general population. All participants in the general population who were healthcare workers (outside of HUG) were excluded. All participants in the healthcare workers group who were not tested were excluded. Participants who had chronic symptoms prior to testing, similar to those listed, were also excluded, considering their symptoms could potentially be due to other causes. All individuals gave consent and the study was approved by the Cantonal Research Ethics Commission of Geneva, Switzerland (protocol numbers 2021–00389 and 2021–00931).

The questionnaire included questions about baseline characteristics, comorbidities, self-rated health, symptoms and evolution of symptoms since testing, current symptoms over the past two weeks, quality of life, functional capacity and productivity using the Sheehan disability scale (Leon et al., 1997) and the work ability index scale (Ilmarinen and Tuomi, 2004). The questionnaire instrument is available as supplementary material. Data was collected using REDCap v11.0.3 and analyzed using the statistical software Stata, version 16.0 (StataCorp). Descriptive analyses and prevalence included percentages, with comparisons using chi-square tests or Fisher's exact test when appropriate. A p-value of < 0.05 was considered as significant. To evaluate the effect of the role of profession (healthcare worker versus non-healthcare worker) and SARS-CoV-2 infection on the outcomes of fatigue, headache, difficulty concentrating, insomnia and exhaustion/burnout, a causal mediation analysis was conducted using the STATA med4way command with a logistic regression model form. The exposure considered in this analysis was the profession (healthcare worker versus non-healthcare worker), the mediator considered in this analysis was SARS-CoV-2 infection, and the confounders based on a directed acyclic graph model were age, sex, time from testing, symptoms at presentation, and COVID-19 vaccination status. Age and time from testing were fixed at their respective mean values, sex at female, symptoms at presentation at symptomatic, and vaccination at fully vaccinated (2 doses or 1 dose with infection, as suggested by the national vaccination guidelines in Switzerland at the time) (Federal Office of Public Health, n.d.). The total excess relative risk corresponds to the effect of the profession (healthcare worker) along with mediation and interaction, the direct excess relative risk due to controlled direct effect represents the effect due to neither mediation nor interaction (profession only), the excess relative risk due to reference interaction represents the portion of effect due to just interaction without mediation, the excess relative risk due to mediated interaction represents the portion of effect due to both mediation and interaction and the excess relative risk due to pure indirect effect represents the portion of effect due to just mediation without interaction.

## 3 Results

The mean age of healthcare workers (n = 3083) was 43.8 years ± 11.0 standard deviation (SD), 72.3 % were women, 43.9 % nursing staff, 19.3 % administrative staff, and 15.9 % physicians. This distribution was in line with the staff distribution at the Geneva University Hospitals. In comparison, the mean age in the general population group (n = 3556) was 44.4 years (SD, 14.4) and 56.5 % were women (Table 1). The median time from infection to follow-up was 244 days (interquartile range IQR 202–400 days) in healthcare workers versus 220 days (IQR 198–344 days) in the general population (p < 0.001).

**Table 1:** Characteristics of healthcare workers and individuals from the general population.*

	Total (n = 6639)	Healthcare workers (n = 3083)	General population (n = 3556)	P-value
	N (%)	N (%)	N (%)	
Age categories				<0.001
below 40	2695(40.6)	1193(38.7)	1502(42.2)	
40–59	3205(48.3)	1699(55.1)	1506(42.4)	
60 and above	739(11.1)	191(6.2)	548(15.4)	

Sex				<0.001
Male	2399(36.2)	854(27.7)	1545(43.5)	
Female	4237(63.8)	2228(72.3)	2009(56.5)	

Test result				0.157
Negative	4256(64.1)	2004(65.0)	2252(63.3)	
Positive	2383(35.9)	1079(35.0)	1304(36.7)	

Smoking				<0.001
Never smoked	3544(53.4)	1750(56.8)	1794(50.5)	
Current smoker	1243(18.7)	550(17.8)	693(19.5)	
Ex-smoker, stopped independently of COVID-19	1673(25.2)	706(22.9)	967(27.2)	
Ex-smoker, stopped because of COVID-19	24(0.4)	8(0.3)	16(0.5)	
Prefer not to answer	151(2.3)	68(2.2)	83(2.3)	

Physical activity				0.104
None	909(13.7)	389(12.6)	520(14.6)	
Partially active	3380(50.9)	1589(51.6)	1791(50.4)	
Regular physical activity	2300(34.7)	1080(35)	1220(34.3)	
Prefer not to answer	46(0.7)	24(0.8)	22(0.6)	

Symptoms at presentation				<0.001
None	1662(25.2)	894(29.5)	768(21.6)	
Pauci-symptomatic	3207(48.7)	1404(46.3)	1803(50.7)	
Had several symptoms	1703(25.9)	729(24)	974(27.4)	
Prefer not to answer	15(0.2)	7(0.2)	8(0.2)	

Regular work activity				<0.001
<30 %	88(1.5)	26(0.8)	62(2.3)	
Between 30 and 49 %	41(0.7)	18(0.6)	23(0.8)	
Between 50 and 79 %	580(10)	371(12)	209(7.7)	
Between 80 and 100 %	5094(87.8)	2666(86.5)	2428(89.2)	

COVID-19 vaccination status				<0.001
Not vaccinated	2189(33)	659(21.4)	1530(43.1)	
Received 2 doses	3172(47.8)	2017(65.4)	1155(32.5)	
Received 1 dose	1225(18.5)	374(12.1)	851(24)	
Prefer not to answer	50(0.8)	33(1.1)	17(0.5)	

Symptoms				
Fatigue	1667(25.1)	1206(39.1)	461(13.0)	<0.001
Headache	729(11.0)	489(15.9)	240(6.7)	<0.001
Difficulty concentrating	583(8.8)	364(11.8)	219(6.2)	<0.001
Insomnia	565(8.5)	423(13.7)	142(4.0)	<0.001
Exhaustion/Burnout	557(8.4)	373(12.1)	184(5.2)	<0.001
Myalgia	465(7.0)	288(9.3)	177(5.0)	<0.001
Arthralgia	368(5.5)	261(8.5)	107(3.0)	<0.001
Dyspnea	370(5.6)	197(6.4)	173(4.9)	0.007
Loss or change in smell	466(7.0)	212(6.9)	254(7.1)	0.672
Loss or change in taste	324(4.9)	144(4.7)	180(5.1)	0.461
Cough	231(3.5)	140(4.5)	91(2.6)	<0.001
Palpitations	184(2.8)	105(3.4)	79(2.2)	0.003
Chest pain	108(1.6)	62(2.0)	46(1.3)	0.021
Stress	460(6.9)	315(10.2)	145(4.1)	<0.001

Functional impairment				0.096
None	5238(78.9)	2423(78.6)	2815(79.2)	
Mild	588(8.9)	259(8.4)	329(9.2)	
Moderate	571(8.6)	272(8.8)	299(8.4)	
Severe	242(3.6)	129(4.2)	113(3.2)	

Co-morbidities				
No comorbidities	3137(47.3)	1545(50.1)	1592(44.8)	<0.001
Obesity or overweight	1088(16.4)	515(16.7)	573(16.1)	0.517
Headache (all types)	815(12.3)	391(12.7)	424(11.9)	0.347
Insomnia	731(11.0)	327(10.6)	404(11.4)	0.327
Hypertension	524(7.9)	185(6.0)	339(9.5)	<0.001
Rheumatological disorder	469(7.1)	169(5.5)	300(8.4)	<0.001
Anxiety	392(5.9)	124(4)	268(7.5)	<0.001
Respiratory disease	300(4.5)	134(4.3)	166(4.7)	0.529
Irritable bowel syndrome	299(4.5)	103(3.3)	196(5.5)	<0.001
Cognitive disorders (attention deficit, memory disorders)	287(4.3)	104(3.4)	183(5.1)	<0.001
Chronic fatigue syndrome	276(4.2)	110(3.6)	166(4.7)	0.025
Depression	266(4.0)	65(2.1)	201(5.7)	<0.001
Anemia	206(3.1)	91(3.0)	115(3.2)	0.508
Hypothyroidism	202(3)	111(3.6)	91(2.6)	0.014
Cardiovascular disease	150(2.3)	44(1.4)	106(3.0)	<0.001
Diabetes	141(2.1)	53(1.7)	88(2.5)	0.033
Dysmenorrhea	142(2.1)	113(3.7)	29(0.8)	<0.001
Chronic pain syndrome/Fibromyalgia	100(1.5)	43(1.4)	57(1.6)	0.487
Immunosuppression	76(1.1)	25(0.8)	51(1.4)	0.017
Hyperthyroidism	56(0.8)	26(0.8)	30(0.8)	0.999
Inflammatory bowel disease	46(0.7)	24(0.8)	22(0.6)	0.434
Thromboembolic disease	43(0.6)	13(0.4)	30(0.8)	0.033
Cancer	30(0.5)	3(0.1)	27(0.8)	<0.001
Renal disease	25(0.4)	8(0.3)	17(0.5)	0.147
Multiple sclerosis	23(0.3)	7(0.2)	16(0.4)	0.123
Psychiatric disorder	13(0.2)	0(0.0)	13(0.4)	0.001
Lupus	13(0.2)	6(0.2)	7(0.2)	0.984

* Pauci-symptomatic was defined in the questionnaire as: “I have symptoms but very few”, as opposed to “I have no symptoms”; “I have several symptoms”, or “Prefer not to answer”.

**Fig. 1:**
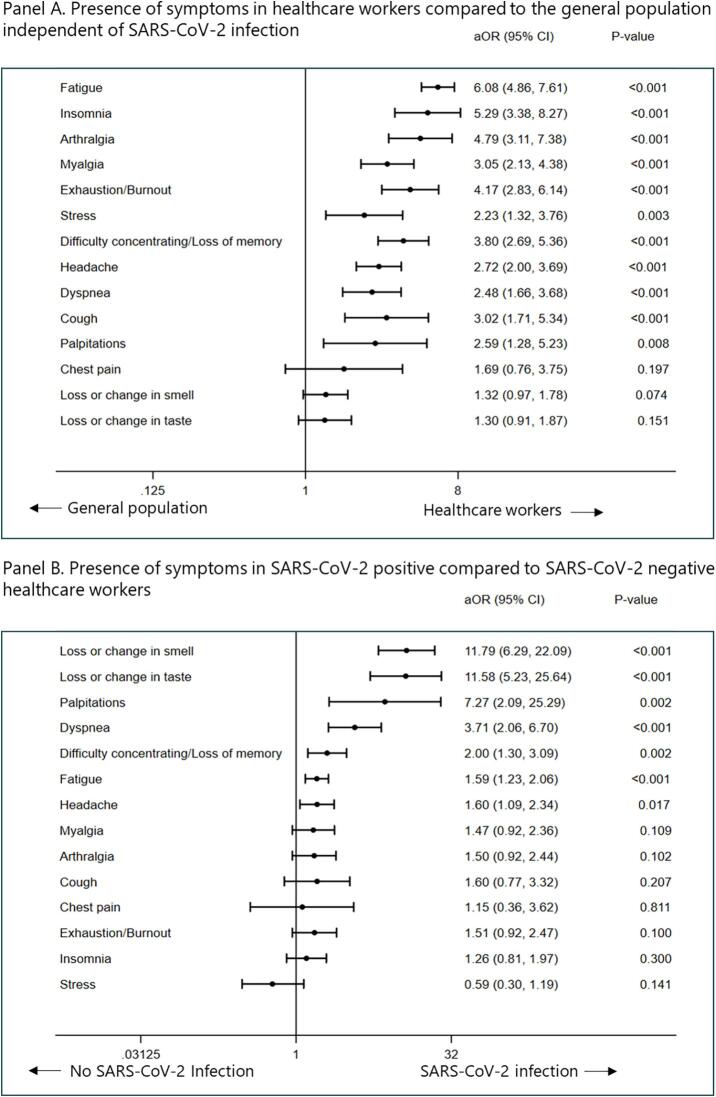
Symptoms prevalence in SARS-CoV-2 infected and non-infected healthcare workers and the general population*, *aOR: adjusted odds ratios; CI: confidence interval, Panel A: Odds ratios were adjusted for test result, age, sex, time from testing, symptoms at presentation, smoking, physical activity, COVID-19 vaccination status, hospitalization, and the following comorbidities present prior to testing: overweight or obese, hypertension, respiratory disease, cardiovascular disease, diabetes, immunosuppression, hypothyroidism, hyperthyroidism, anemia, headache (migraine or tension headache), cognitive disorders (attention deficit disorder or memory disorder), sleeping disorder, anxiety, depression, any psychiatric condition, irritable bowel syndrome, chronic pain syndrome, fibromyalgia, and chronic fatigue, Panel B: Odds ratios were adjusted for age, sex, time from testing, symptoms at presentation, smoking, physical activity, COVID-19 vaccination status, hospitalization, and the following comorbidities present prior to testing: overweight or obese, hypertension, respiratory disease, cardiovascular disease, diabetes, immunosuppression, hypothyroidism, hyperthyroidism, anemia, headache (migraine or tension headache), cognitive disorders (attention deficit disorder or memory disorder), sleeping disorder, anxiety, depression, any psychiatric condition, irritable bowel syndrome, chronic pain syndrome, fibromyalgia, and chronic fatigue.

Symptoms prevalence was higher in healthcare workers than in the general population independently of SARS-CoV-2 infection (Table 1). Fatigue, headache, difficulty concentrating/loss of memory, exhaustion/burnout, arthralgia, myalgia, and insomnia were among the most prominent symptoms in healthcare workers compared to the general population (Table 1 and Fig. 1, panel A). SARS-CoV-2 infection was associated with loss or change in smell, loss or change in taste, palpitations, dyspnea, difficulty concentrating/loss of memory, fatigue and headache in healthcare workers (Fig. 1, panel B). Mediation analysis shows an association between exposure (healthcare worker) and the different outcomes, with an excess relative risk due to controlled direct effect without mediation nor interaction. There is also an excess relative risk due to pure indirect effect (SARS-CoV-2 infection). Results are shown in Table 2.

**Table 2:** Coefficients of mediation analysis between exposure (healthcare workers) and outcomes (fatigue, headache, difficulty concentrating, exhaustion/burnout, insomnia), considering mediators and interactions.*

	Fatigue	Headache	Difficulty concentrating	Insomnia	Exhaustion/ Burnout
Total excess relative risk	3.21 (2.59;3.83)	1.37 (0.92;1.83)	1.14 (0.69;1.59)	2.46 (1.68;3.25)	1.44 (0.91;1.97)
Excess relative risk due to controlled direct effect	3.04 (2.20–3.87)	1.04 (0.43;1.65)	1.33 (0.62;2.04)	2.04(1.05;3.03)	1.06 (0.34;1.78)
Excess relative risk due to reference interaction	0.11 (−0.34;0.57)	0.31 (−0.05;0.66)	−0.29 (−0.68;0.11)	0.39(−0.01;0.92)	0.35 (−0.05;0.75)
Excess relative risk due to mediated interaction	−0.01 (−0.06;0.04)	−0.03 (−0.07;0.01)	0.03 (−0.01;0.08)	−0.04 (−0.10;0.02)	−0.04 (−0.09; 0.01)
Excess relative risk due to pure indirect effect	0.07 (0.04;0.10)	0.06 (0.02;0.09)	0.07 (0.03;0.10)	0.07(0.03;0.11)	0.07 (0.03;0.11)

* The total excess relative risk corresponds to the effect of the profession along with mediation and interaction, the direct excess relative risk due to controlled direct effect represents the effect due to neither mediation nor interaction, the excess relative risk due to reference interaction represents the portion of effect due to just interaction without mediation, the excess relative risk due to mediated interaction represents the portion of effect due to both mediation and interaction and the excess relative risk due to pure indirect effect represents the portion of effect due to just without interaction.

Overall, 61.6 % of healthcare workers reported working at 95–100 % of their functional capacity versus 77.2 % of individuals in the general population (unadjusted and adjusted p-values < 0.001). The odds of having moderate to severe functional impairment was higher in healthcare workers compared to the general population (aOR 2.28; 1.76–2.96), as well as in SARS-CoV-2 infected versus non-infected healthcare workers (aOR 3.81; 2.59–5.60).

## 4 Discussion

Healthcare workers suffer from a high prevalence of symptoms associated with the pandemic toll in general and with the effects of SARS-CoV-2 infection more specifically. These symptoms include fatigue, headache, difficulty concentrating, insomnia, and exhaustion/burnout among others. A mediation analysis shows that being a healthcare worker is directly associated with a higher likelihood of these symptoms, even after considering potential mediator and interaction effects. Functional impairment is also more prevalent in healthcare workers compared to the general population, and even more so in SARS-CoV-2 positive compared to SARS-CoV-2 negative healthcare workers. Healthcare associated infections account for 4.3 % all COVID-19 cases in Geneva (Mongin et al., 2019). Studies suggested that healthcare workers were at risk of burnout and mental health issues during the first pandemic wave (Pappa et al., 2020); (Carmassi et al., 2020), and healthcare workers were generally considered at a higher risk of fatigue, insomnia and burnout even prior to the pandemic (Gates et al., 2018 Sep 21). In this current COVID-19 pandemic, smaller studies showed that 45 % of 138 healthcare workers had persistent symptoms after their SARS-CoV-2 infection (Gaber et al., 2021 Jun 16), and 26.5 % and 13.5 % of 260 healthcare workers in a Swiss hospital reported persistent symptoms at 3 and 12 months after the infection (Martinez et al., 2021). Additionally, healthcare workers were more likely to be vaccinated in this study, and some symptoms including headaches have been associated with vaccination (Göbel et al., 2021). To note however that headaches were associated to date with the ChAdOx1 nCoV-19 (AZD1222) vaccine which has not been used in Switzerland (Göbel et al., 2021). This current study highlights both the differentially more important pandemic burden on healthcare workers compared to the general population and the added direct effect of SARS-CoV-2 infection, with a potential functional impairment that could develop into long-term overall reduced work capacity. Limitations include potential ascertainment bias similarly to questionnaires in general (Althubaiti, 2016 May), with the subjective rating of self-reported symptoms and a potentially higher health literacy in healthcare workers. The study lacked differentiation between frontline and non-frontline workers but showed an overall prevalence of symptoms in healthcare workers. Additionally, this study being a cross-sectional design, it is difficult to assess a causal inference. Using the mediation analysis mitigates this limitation but does not remove it completely. Looking forward, different variants and an ongoing pandemic may induce different stress levels to be taken into consideration (Temsah et al., 2022). Health systems cannot function without healthcare workers, and it is important to pay special attention and provide additional care and support to our staff in order to ensure the proper recovery and wellbeing of those who have been on the frontlines since the beginning of the pandemic, who have to face new potential waves and continue caring for all patients including and beyond COVID-19.

## Declaration of Competing Interest

The authors declare that they have no known competing financial interests or personal relationships that could have appeared to influence the work reported in this paper.

## References

[b0005] Althubaiti A. (2016). Information bias in health research: definition, pitfalls, and adjustment methods. J Multidiscip Healthc..

[b0010] Bergwerk M., Gonen T., Lustig Y., Amit S., Lipsitch M., Cohen C., Mandelboim M., Levin E.G., Rubin C., Indenbaum V., Tal I., Zavitan M., Zuckerman N., Bar-Chaim A., Kreiss Y., Regev-Yochay G. (2021). Covid-19 Breakthrough Infections in Vaccinated Health Care Workers. N Engl J Med.

[b0015] Carmassi C., Foghi C., Dell'Oste V., Cordone A., Bertelloni C.A., Bui E., Dell'Osso L. (2020). PTSD symptoms in healthcare workers facing the three coronavirus outbreaks: What can we expect after the COVID-19 pandemic. Psychiatry Research.

[b0020] Federal Office of Public Health, Coronavirus: Vaccination. Switzerland https://www.bag.admin.ch/bag/en/home/krankheiten/ausbrueche-epidemien-pandemien/aktuelle-ausbrueche-epidemien/novel-cov/impfen.html#-995735508 [Accessed July 18, 2021].

[b0025] Gaber T.A.K., Ashish A., Unsworth A. (2021). Persistent post-covid symptoms in healthcare workers. Occup Med (Lond)..

[b0030] Gates M., Wingert A., Featherstone R., Samuels C., Simon C., Dyson M.P. (2018). Impact of fatigue and insufficient sleep on physician and patient outcomes: a systematic review. BMJ Open..

[b0035] Gholami M., Fawad I., Shadan S., Rowaiee R., Ghanem HedaietAllah, Hassan Khamis A., Ho S.B. (2021). COVID-19 and healthcare workers: A systematic review and meta-analysis. International Journal of Infectious Diseases.

[b0040] Göbel C.H., Heinze A., Karstedt S., Morscheck M., Tashiro L., Cirkel A., Hamid Q., Halwani R., Temsah M.-H., Ziemann M., Görg S., Münte T., Göbel H. (2021). Headache Attributed to Vaccination Against COVID-19 (Coronavirus SARS-CoV-2) with the ChAdOx1 nCoV-19 (AZD1222) Vaccine: A Multicenter Observational Cohort Study. Pain Ther.

[b0045] Havervall S., Rosell A., Phillipson M., Mangsbo S.M., Nilsson P., Hober S., Thålin C. (2021). Symptoms and Functional Impairment Assessed 8 Months After Mild COVID-19 Among Health Care Workers. JAMA..

[b0050] Ilmarinen J., Tuomi K. (2004). Past, present and future of work ability. People and Work Research Reports. Finnish Institute of Occupational Health, Helsinki.

[b0055] National Institutes of Health (NIH). When COVID-19 Symptoms Linger New NIH initiative seeks to understand why some people continue to have symptoms long after recovery. Last updated August 13, 2021 https://covid19.nih.gov/news-and-stories/when-COVID-19-symptoms-linger [Access August 22, 2021].

[b0060] Leon A.C., Olfson M., Portera L., Farber L., Sheehan D.V. (1997). Assessing psychiatric impairment in primary care with the Sheehan Disability Scale. Int J Psychiatry Med..

[b0065] Lopez-Leon S., Wegman-Ostrosky T., Perelman C., Sepulveda R., Rebolledo P.A., Cuapio A., Villapol S. (2021). More than 50 long-term effects of COVID-19: a systematic review and meta-analysis. Sci Rep..

[b0070] Martinez A.E., Banderet F., Labhardt N.D., Battegay M. (2021). Long-term outcome after SARS-CoV-2 infection in healthcare workers: a single centre cohort study. Swiss Med Wkly..

[b0075] Maunder R.G., Lancee W.J., Balderson K.E., Bennett J.P., Borgundvaag B., Evans S., Fernandes C.M., Goldbloom D.S., Gupta M., Hunter J.J., McGillis Hall L., Nagle L.M., Pain C., Peczeniuk S.S., Raymond G., Read N., Rourke S.B., Steinberg R.J., Stewart T.E., VanDeVelde-Coke S., Veldhorst G.G., Wasylenki D.A. (2006). Long-term psychological and occupational effects of providing hospital healthcare during SARS outbreak. Emerg Infect Dis..

[b0080] Mehta S., Machado F., Kwizera A., Papazian L., Moss M., Azoulay É., Herridge M. (2021). COVID-19: a heavy toll on health-care workers. The Lancet Respiratory Medicine.

[b0085] Mongin D, Catho G, Iten A, Harbarth S, Courvoisier DS. Incidence of healthcare-associated coronavirus disease 2019 (COVID-19) in the state of Geneva. Infect Control Hosp Epidemiol. 2021 Oct 25:1-3. doi: 10.1017/ice.2021.453. Epub ahead of print. PMID: 34689854; PMCID: PMC8593366. 10.1017/ice.2021.453 PMC8593366 34689854

[b0090] Nehme M, Braillard O, Alcoba G, Aebischer Perone S, Courvoisier D, Chappuis F, Guessous I. COVID-19 Symptoms: Longitudinal Evolution and Persistence in Outpatient Settings. Ann Intern Med. 2020 Dec 8:M20-5926. doi: 10.7326/M20-5926. Epub ahead of print. PMID: 33284676; PMCID: PMC7741180. 10.7326/M20-5926 PMC7741180 33284676

[b0095] Nehme M, Braillard O, Chappuis F, Courvoisier DS, Guessous I. Prevalence of Symptoms More Than Seven Months After Diagnosis of Symptomatic COVID-19 in an Outpatient Setting. Ann Intern Med. 2021 Jul 6. doi: 10.7326/M21-0878. Epub ahead of print. PMID: 34224254. 10.7326/M21-0878 PMC8280535 34224254

[b0100] Pappa S, Ntella V, Giannakas T, Giannakoulis VG, Papoutsi E, Katsaounou P. Prevalence of depression, anxiety, and insomnia among healthcare workers during the COVID-19 pandemic: A systematic review and meta-analysis. Brain Behav Immun. 2020 Aug;88:901-907. doi: 10.1016/j.bbi.2020.05.026. Epub 2020 May 8. Erratum in: Brain Behav Immun. 2021 Feb;92:247. PMID: 32437915; PMCID: PMC7206431. 10.1016/j.bbi.2020.05.026 PMC7206431 32437915

[b0105] Stringhini S., Zaballa M.-E., Pullen N., de Mestral C., Perez-Saez J., Dumont R., Picazio A., Pennacchio F., Dibner Y., Yerly S., Baysson H., Vuilleumier N., Balavoine J.-F., Bachmann D., Trono D., Pittet D., Chappuis F., Kherad O., Kaiser L., Azman A.S., Alber V., Arm-Vernez I., Bachmann D., Bachmann D., Baggio S., Monteiro G.B., Baysson H., Bleich P., Boissel I., Collombet P., Courvoisier D., Couson P., Davidovic A., Deiri C., Del Rio D., de Mestral C., De Ridder D., D’ippolito P., Duc J., Eckerle I., El Merjani N., Ferniot G., Flahault A., Francioli N., Frangville M., Garande C., Gétaz L., Giraldo P., Golaz F., Guérin J., Haboury L., Harnal S., Javet V., Kaiser L., Laboulais A., Lamour G., Lefebvre X., Lescuyer P., Loizeau A.J., Lombard F.-B., Lorthe E., Martinez C., Massiha K., Metral-Boffod L., Meyer B., Mostaguir K., Nehme M., Noël N., Oederlin N., Petrovic D., Piumatti G., Portier J., Poulain G., Pugin C., Rakotomiaramanana B., Randrianandrasana Z.F., Richard A., Richard V., Rodriguez-Velazquez S., Salzmann-Bellard L., Thorens L., Torroni S., Vidonne D., Violot G., Waldmann Z., Will M., Wisniak A., Guessous I. (2021). Large variation in anti-SARS-CoV-2 antibody prevalence among essential workers in Geneva, Switzerland. Nat Commun.

[b0110] Temsah M.H., Alenezi S., Alarabi M., Aljamaan F., Alhasan K., Assiri R., Bassrawi R., Alshahrani F., Alhaboob A., Alaraj A., Alharbi N.S., Alrabiaah A., Halwani R., Jamal A., Abdulmajeed N., Alfarra L., Almashdali W., Al-Eyadhy A., AlZamil F., Alsubaie S., Barry M., Memish Z.A., Al-Tawfiq J.A. (2022). Healthcare Workers’ SARS-CoV-2 Omicron Variant Uncertainty-Related Stress, Resilience, and Coping Strategies during the First Week of the World Health Organization’s Alert. Int J Environ Res Public Health..

