# Supplementary material for: The pandemic toll and post-acute sequelae of SARS-CoV-2 in healthcare workers at a Swiss University Hospital
Source: Prev Med Rep. 2022 Jul 8;29:101899. doi: 10.1016/j.pmedr.2022.101899 (PMC9263685; doi:10.1016/j.pmedr.2022.101899)
Supplement: Supplementary Data 1 [file mmc1.docx]

| **Supplement 1. Questionnaire instrument** | | |  | |
| --- | --- | --- | --- | --- |
| Occupation (Healthcare workers) | Administrative personnel | | | |
|  | Logistics | | | |
|  | Social workers | | | |
|  | Technical services | | | |
|  | Nursing personnel | | | |
|  | Physicians | | | |
|  | Medical technicians | | | |
|  | Medical therapists (physiotherapy, occupational therapy) | | | |
|  | Sanitation and restauration staff | | | |
| Occupation (General population) | Salaried  Retired  Student  Independent  Looking after home/family  Unemployed  Disability  Other | | | |
|  |  |  |  |  |
|  |  |  |  |  |
|  |  |  |  |  |
|  |  |  |  |  |
|  |  |  |  |  |
|  |  |  |  |  |
| Profession (General population) | Unskilled workers (manual labor, craftsmen, traders, farmers, employees without specific training)  Skilled workers (employees with specific training)  Highly skilled workers (employees with intermediate training)  Professional-Managers (company managers with more than 10 employees, individuals with a profession requiring university training)  Independent (consultants, were independent or were company managers with fewer than 10 employees)  Other  Prefer not to answer | | | |
|  |  |  |  |  |
|  |  |  |  |  |
|  |  |  |  |  |
|  |  |  |  |  |
| Work in healthcare setting (General population) | Yes (Hospital, clinic, nursing home, homecare)  No | | | |
| Smoking status | I have never smoked  I am a current smoker  I am an ex-smoker, but stopped prior to my SARS-CoV-2 infection  I am an ex-smoker, but stopped after my SARS-CoV-2 infection | | | |
| Physical activity | I do not do any physical activity  I am partially active  I am completely active | | | |
| Compared to before the test date, your physical activity is: | The same  Less  More | | | |
| Do you suffer from any of the following conditions (multiple answers possible) | None  Overweight  Sleep disorders  Migraine  Hypertension  Anxiety  Irritable bowel syndrome  Depression  Chronic fatigue syndrome  Respiratory disease  Other arthritic disorder (specify)  Tension headache  Tendinitis  Obesity  Anemia  Attention disorders  Memory disorders  Hypothyroidism  Other digestive disorder (specify)  Cardiovascular disease  Other type of headache (specify)  Diabetes  Chronic pain syndrome  Immunosuppression  Deep vein thrombosis  Dysmenorrhea  Hyperthyroidism  Rheumatoid arthritis  Other neurologic disorder (specify)  Fibromyalgia  Ulcerative colitis  Multiple sclerosis  Cancer  HIV  Other psychiatric disorder (specify)  Reactive arthritis  Renal disease  Ankylosing spondylitis  Crohn disease  Lupus  Sjogren disease  Cirrhosis | | | |
| If condition checked, specify | [This condition] is new since my test date  I suffer from [this condition] since before my test date  Prefer not to answer | | | |
| Did you have COVID-19 compatible symptoms at time of testing? | Yes, I had symptoms  Yes, but very few symptoms  No, I did not have symptoms  Prefer not to answer | | | |
| After your test date, how did your symptoms evolve? | I never had symptoms  My symptoms disappeared  My symptoms are fluctuating  My symptoms are constant  Other  Prefer not to answer | | | |
| What was or has been the duration of symptoms since they started? | 0-10 days  11-20 days  21-30 days  1-2 months  2-3 months  3-4 months  4-5 months  5-6 months  6-7 months  8-9 months  9-10 months  10-11 months  11-12 months  More than 12 months  Do not know | | | |
| After your laboratory confirmed test at the Geneva University Hospitals, did you have any nasopharyngeal swab (RT-PCR or antigenic test) positive for COVID-19? | Yes  No | | If yes, date of test | |
| Have you had any serological testing for COVID-19 | Yes  No | | If yes, date of test and result | |
| Have you been vaccinated against SARS-CoV-2 | Yes, I received 1 dose  Yes, I received 2 doses  No  Prefer not to answer | | If yes, date of each dose | |
| Which type of vaccine did you receive | Comirnaty® (BNT162b2) vaccine of Pfizer/BioNTech  (mRNA-1273) vaccine of Moderna  Vaxzevria (previously COVID-19 vaccine of Oxford/AstraZeneca)  Sinopharm BIBP vaccine of China National Pharmaceutical Group  Sputnik V vaccine of Gamaleya Research Institute of Epidemiology and Microbiology  Janssen Vaccine of Johnson&Johnson  Do not know  Prefer not to answer  Other | | | |
| Did you receive any of the following treatments in the first 21 days after your test date? (multiple answers possible) | None  Paracetamol  Non-steroidal anti-inflammatory medication  Antiviral (ritonavir, lopinavir, remdesivir etc.)  Dexamethasone, prednisone or other steroid  Monoclonal antibodies (Tocilizumab etc.)  Inhaled spray (Seretide®, Ventolin®, Symbicort®, Spiriva®, Atrovent®, Bricanyl®, Dospir®, etc.)  Nasal spray (Nasonex®, Rhinomer® etc.)  Hydroxychloroquine (Plaquenil®)  Ivermectin  Anticoagulation (Lovenox®, Arixtra® etc.)  Homeopathic treatment  Zinc  Vitamin C  Vitamin D  Other  Prefer not to answer | | | |
| Have you been hospitalized since your test date? | Yes  No  Prefer not to answer | | If yes, was the hospitalization related to COVID-19 and date of hospitalization | |
| Have you seen a physician or healthcare professional in relation to your symptoms? (multiple answers possible) | Yes, my primary care physician  Yes, I have been to the emergency room  Yes, I have seen another specialist, physician or healthcare professional (specify)  No | | If yes, how many times for each answer and is this a new follow-up since your test date? | |
| In the past 2 weeks, which of the following symptoms have you experienced, even if fluctuating? (multiple answers possible) | Fatigue  Headache  Change in smell  Mental exhaustion  Myalgia  Dyspnea  Difficulty concentrating  Insomnia  Stress  Change in taste  Loss of memory  Paresthesia  Arthralgia  Neck pain  Anxiety  Cough  Sadness  Loss of smell  Palpitations  Dizziness  Back pain  Throat pain  Diarrhea  Nausea  Abdominal pain  Loss of taste  Hair loss  Lack of equilibrium  Lack of appetite  Chest pain  Constipation  Generalized pain  Fever > 38 C  Rash  Malaise  Vomiting  Toe pain or redness  Finger pain or redness  Other  Prefer not to answer | | | |
| If symptom checked, did you have this symptom regularly (most days) prior to the test? | Yes  No  Prefer not to answer | | | |
| Current symptom intensity | Mild  Moderate  Severe | | | |
| Current symptom frequency | Never  Rarely  Often  Always | | | |
| If fatigue, specify | Normal activity, no limitations in daily activity  Limited activity but capable to do light work (office work, cleaning)  Limited activity but <50% in bed during the day  >50% in bed, but not bedbound  Bedbound | | | |
| If fatigue, | Do you have problems with tiredness? | | Yes  No | |
|  | Do you need to rest more? | | Yes  No | |
|  | Do you feel sleepy or drowsy? | | Yes  No | |
|  | Do you have problems starting things? | | Yes  No | |
|  | Do you lack energy? | | Yes  No | |
|  | Do you have less strength in your muscles? | | Yes  No | |
|  | Do you feel weak? | | Yes  No | |
|  | Do you have difficulty concentrating? | | Yes  No | |
|  | Do you make slips of the tongue when speaking? | | Yes  No | |
|  | Do you find it more difficult to find the right word? | | Yes  No | |
|  | Is your memory as good as usual? | | Yes  No | |
| If dyspnea, specify | Dyspnea only with strenuous exercise  Dyspnea when hurrying or walking up a slight hill  Has to stop for breath when walking at own pace on flat surface  Stops for breath after walking 90 meters or after a few minutes  Too breathless to leave house or breathless when dressing | | |  |
| If insomnia, specify (over the past 2 weeks) | Do you have difficulty falling asleep? | | None  Mild  Moderate  Severe  Very severe | |
|  | Do you have difficulty staying asleep? | | None  Mild  Moderate  Severe  Very severe | |
|  | Do you have problems waking up too early? | | None  Mild  Moderate  Severe  Very severe | |
|  | How satisfied/dissatisfied are you with your current sleep pattern? | | Very satisfied  Satisfied  Moderately satisfied  Dissatisfied  Very dissatisfied | |
|  | How noticeable to others do you think your sleep problem is in terms of impairing the quality of your life? | | Not at all noticeable  A little  Somewhat noticeable  Very much noticeable | |
|  | How worried/distressed are you about your current sleep problem? | | Not at all worried  A little  Somewhat worried  Very much worried | |
|  | To what extent do you consider your sleep problem to interfere with your daily functioning currently? | | Not at all interfering  A little  Somewhat interfering  Very much interfering | |
| If symptom checked, specify: | Your symptoms have disrupted your work/school work | | Scale:  0 Not at all – 10 Extremely | |
|  | Your symptoms have disrupted your social life/leisure activities | | Scale:  0 Not at all – 10 Extremely | |
|  | Your symptoms have disrupted your family life/home responsibilities | | Scale:  0 Not at all – 10 Extremely | |
|  | On how many days in the last week did your symptoms cause you to miss school or work or leave you unable to carry out your normal daily responsibilities? | | | |
|  | On how many days in the last week did you feel so impaired by your symptoms that even though you went to school or work, your productivity was reduced? | | | |
| Work ability index | Assume that your work ability at its best has a value of 10 points. How many points would you give your current work ability?  (0 means that you currently cannot work at all; 10 work ability at its best) | | | |
| In general, would you say your health is currently | Excellent, very good, good, fair, poor | |  | |
| Have you ever been followed by a psychiatrist for any mental health condition prior to March 2020 (including depression, anxiety, sleeping disorders, suicidal ideations, fear of others, trauma, other problems) | |  | Yes  No | |
